# Acute Treatment With Fingolimod Does Not Confer Long-Term Benefit in a Mouse Model of Intracerebral Haemorrhage

**DOI:** 10.3389/fphar.2020.613103

**Published:** 2021-01-08

**Authors:** Andrea C. Diaz Diaz, Jennifer A. Shearer, Kyle Malone, Christian Waeber

**Affiliations:** ^1^ School of Pharmacy, University College Cork, Cork, Ireland; ^2^ Department of Pharmacology and Therapeutics, University College Cork, Cork, Ireland

**Keywords:** haemorrhagic stroke, immunomodulator, lymphocytes, sex differences, sphingosine 1-phosphate

## Abstract

Intracerebral haemorrhage (ICH) has no specific treatment, but accounts for up to 15% of all strokes and has the highest mortality. Fingolimod (FTY720) is an immunomodulator approved for the management of multiple sclerosis, with abundant evidence of efficacy in experimental ischemic stroke, and more limited evidence in experimental ICH. The goal of this study was to confirm the efficacy of fingolimod in experimental ICH using rigorous and statistically well-powered studies. ICH was induced in C57BL/6JOlaHsd male and female mice by intrastriatal bacterial collagenase injection. Fingolimod (0.5 mg/kg) or saline was administered intraperitoneally after 0.5, 24 and 72 h, in a randomized and blinded manner. Functional improvement with cylinder, wire hanging, and foot fault tests was evaluated one and two weeks later. Lesion volume and hemispheric atrophy were quantified at the 14-day endpoint. There was a higher mortality in saline-treated females compared to fingolimod-treated females and saline-treated males. There was no treatment- or gender-related difference in the behavioural tests. Histological outcome measures did not differ between any of the groups. These results, contrasting with those of previous studies of fingolimod in experimental ICH, emphasize the importance of rigorous testing of this agent in models more representative of the clinical situation.

## Introduction

Ischemic and haemorrhagic strokes are among the leading causes of death and long-term disability worldwide (Benjamin et al., 2019). Although the incidence of ischemic stroke is much higher than that of haemorrhagic stroke, both conditions cause a similar number of deaths worldwide (Katan and Luft, 2018). This may be caused in part by the fact that the only specific therapeutic agent currently available for stroke (recombinant tissue plasminogen activator, or rtPA) can only be used in ischemic stroke, and there is no specific treatment for haemorrhagic stroke.

Stroke pathophysiology is characterised by a succession of short-lasting processes (e.g., excitotoxicity, oxidative stress, blood brain barrier breakdown, apoptotic cell death) (Barone and Feuerstein, 1999). Furthermore, many potential drugs aim at salvaging the penumbra, a reversibly injured brain tissue surrounding the ischemic core that progressively shrinks within hours after the ischemic event (Liu et al., 2010). Therapeutic interventions, such as neuroprotection or recanalization, targeting these processes or brain regions are therefore bound to have a narrow treatment time window. In contrast, post-stroke inflammatory processes operate over days, even weeks, and the efficacy of interventions targeting them may be less critically dependent on defining an optimal therapeutic window (Barone and Feuerstein, 1999). This premise, taken together with the observation that the inflammatory response to ischemic and haemorrhagic stroke is generally similar, has led to various anti-inflammatory agents being tested both in experimental stroke and in stroke patients (Kleinig and Vink, 2009).

While the aetiology of stroke and multiple sclerosis differ, some pathophysiological processes (e.g., role of the adaptive immune system and of inflammation) play a role in both conditions (Lopes Pinheiro et al., 2016), suggesting that they may respond to the same pharmacological interventions. The immunomodulator fingolimod (Gilenya, Novartis), which has been approved for the management of relapsing multiple sclerosis, has garnered interest for the treatment of stroke. A first meta-analysis of the effects of this agent in experimental ischemic stroke was published in 2012 (Liu et al., 2012), and an updated meta-analysis was published very recently, that included a total of 17 articles (Dang et al., 2020). The methodological quality of studies was evaluated using a 10-item checklist developed by the Collaborative Approach to Meta-Analysis and Review of Animal Data in Experimental Stroke (CAMARADES) (Sena et al., 2007). While the meta-analysis concluded that fingolimod reduces infarct volume and improves neurobehavioral outcomes, it also noted that the median quality score of the included articles was 6 out of 10 (IQR, 4–8).

At the clinical level, fingolimod was tested in three open-label studies in patients with ischemic stroke, alone (Fu et al., 2014b) or in combination with rtPA (Zhu et al., 2015; Tian et al., 2018) (including a total of 22, 47 and 46 patients, respectively). To the best of our knowledge, the results of only one open label study in 23 patients with haemorrhagic stroke have been published (Fu et al., 2014a). These four clinical studies, suggesting that fingolimod administration to stroke patients is generally safe and associated with an improved outcome, need to be confirmed in larger randomized double-blind trials.

The current body of preclinical and clinical literature highlights the relative predominance of papers published on the effect of fingolimod on ischemic stroke. Indeed, the effects of fingolimod in experimental intracerebral haemorrhage (ICH) were only the subject of three published studies (Rolland et al., 2011; Rolland et al., 2013; Lu et al., 2014) (Table 1). Similar effects of siponimod, a selective S1P_1_ sphingosine 1-phosphate (S1P) receptor modulator, were also recently published (Bobinger et al., 2019) (fingolimod recognizes S1P_3_, S1P_4_ and S1P_5_ receptors in addition to S1P_1_ (Brinkmann, 2009)). The evidence for an effect of fingolimod in ICH remains therefore relatively weaker than in ischemic stroke. The goal of our studies was to test the effects of fingolimod in a mouse model of ICH using a properly powered study, using both male and female mice, and to report our findings following the ARRIVE (Animal Research: Reporting *In Vivo* Experiments) guidelines (Kilkenny et al., 2010).

**TABLE 1:** Summary of previous studies of S1P receptor modulators in experimental intracerebral haemorrhage.

	Rolland et al., 2011	Rolland et al., 2013		Lu et al., 2014	Bobinger et al., 2019
Drug, dose, route	Fingolimod, 1 mg/kg, i.p	Fingolimod, 1 mg/kg, i.p		Fingolimod, 0.5 mg/kg, i.p	• Siponimod, 0.3 mg/kg, i.p. • Siponimod, 3 mg/kg, i.p
Treatment regimen	1 h after surgery	• 1 h after surgery. • 1 h after surgery and once daily on the following two days		30 min after surgery and once daily on the following two days	• 30 min after surgery. • 30 min after surgery and once daily on the following two days
Sex, strain, species	CD-1 mice (sex not reported)	Male CD-1 mice	Male Sprague-Dawley rats	Male CD1 mice	Male C57BL/6 mice
Model	Collagenase	• collagenase. • autologous blood	Collagenase	Collagenase	Collagenase
Group sizes	5 or 10	7	9–10	10	
**Histological outcome**					
Edema	Wet-dry: *p* < 0.05 (D1 and D3)	Wet-dry: *p* < 0.05 (D1 and D3)		Wet-dry: *p* < 0.05, D3 (n = 5)	• MRI: *p* = 0.02 at D3 (multiple dosage). • Wet-dry: *p* = 0.022, *p* = 0.0013, *p* = 0.04 for single and multiple 0.3, and multiple 3 mg/kg doses
Atrophy/tissue loss			*p* < 0.05 at 10 weeks	*p* < 0.01, D14	
Apoptotic cells				*p* < 0.05, D3	
Behavioral outcome					
Composite neuroscore	*p* < 0.05 D1 and D3	*p* < 0.05 D1 and D3		*p* < 0.05, D3	No effect at D1 *p* = 0.03 at D3
Wire hanging	*p* < 0.05 (D1) n.s. (D3)	*p* < 0.05 (D1)* n.s. (D3)*		*p* < 0.05, D3 and D14	
Beam balance	*p* < 0.05 (D1). n.s. (D3)	*p* < 0.05 (D1)* n.s. (D3)*			
Forelimb use asymmetry		n.s at D1 and D3*			
Corner test		*p* < 0.05 D1 and D3			
Paw placement		*p* < 0.05 D1 and D3	*p* < 0.05 D1 and D2. No effect at D3 and 10 weeks		
**Weight loss**				*p* < 0.01 (D1), *p* < 0.05 (D3)	*p* = 0.036 (multiple 0.3 mg/kg). *p* = 0.048 (multiple 3 mg/kg)
**Survival**				No effect	*p* = 0.037 (multiple 0.3 mg/kg doses)

* Test only performed in the collagenase model.

There are two main rodent ICH models: direct intraparenchymal blood injection and injection of bacterial collagenase to weaken the basal lamina of brain capillaries, leading to spontaneous haemorrhage; the pros and cons of both have been reviewed (Rolland et al., 2013; Kathirvelu and Carmichael, 2015). We chose the collagenase model in mice to enable comparison of our results with those obtained in previous studies (Rolland et al., 2011; Rolland et al., 2013; Lu et al., 2014; Bobinger et al., 2019) (one of those found that fingolimod had beneficial effects on both models (Rolland et al., 2013)). We evaluated whether fingolimod reduces the lesion size in a chronic model of intracerebral haemorrhage 14 days after stroke, as well as functional recovery over time. To lower the number of comparisons, a single dose of fingolimod was chosen (0.5 mg/kg, intraperitoneally) because it showed the largest effect size across various ischemic stroke studies (Dang et al., 2020) and was found to be effective in an experimental ICH study (Lu et al., 2014).

## Methods

### Experimental Design and Animals

The study was performed following approval by the Animal Experimentation Ethics Committee (AEEC) at University College Cork, under a project authorization by the Irish Health Products Regulatory Authority (HPRA; AE19130/P042), and in accordance with the National Institute of Health Guide for Care and Use of Laboratory Animals (National Research Council, 2011). The study was carried out and its results reported according to the ARRIVE guidelines (Kilkenny et al., 2010). Because this study was part of a larger Health Research Board-funded project including both ischemic and haemorrhagic stroke studies, an overall group size calculation for the whole project was performed at the time of grant preparation, using the overall fingolimod effect size (on histological and behavioural improvement) and functional outcome variability from a previously published meta-analysis (Liu et al., 2012), with a significance level set at *α* = 0.05 and beta cut-off of 20%. A group size of 15 mice was set for all experiments in the funded project, acknowledging that this might lead to some type I errors.

All mice were acclimatized for at least one week before any procedure took place. Mice were group-housed in sets of 3 or 4 (with exception of mice that were isolated due to aggressive behaviour and fighting) in open-top cages with aspen wood chip bedding, plus shredded paper and additional environmental enrichment. They had free access to food and water and the facility had a 12 h light/dark cycle.

**FIGURE 1:**
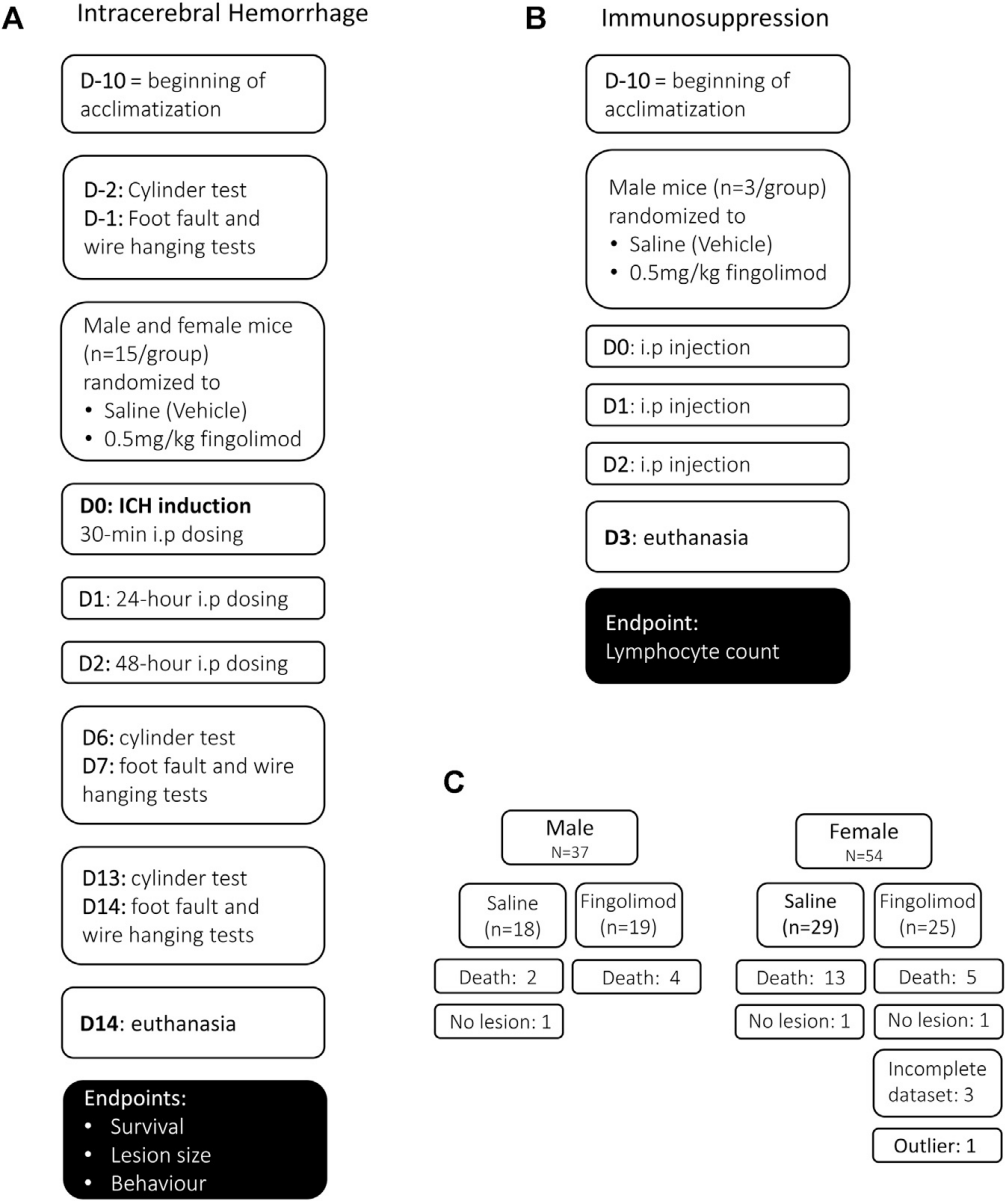
Experimental design for the intracerebral haemorrhage study **(A)**, the immunosuppression study **(B)** and mouse allocation **(C)**. **(A)** In the intracerebral haemorrhage study, baseline behaviour was assessed once mice had been acclimatized. Mice were randomized to the two treatment groups before intracerebral haemorrhage (ICH) was induced, and mice were treated 30 min post-operatively, followed by 24 and 48 h i.p. dosing. Behavioural assessment was carried out at 6, 7 days and 13, 14 days. **(B)** In the immunosuppression study, mice that had been acclimated for at least 10 days received three doses of either saline or fingolimod in a randomized and blinded manner. Blood was collected on day 3 for lymphocyte count. **(C)** A total of 91 mice were used in the ICH study; 24 mice died before the 14-day experimental endpoint, and three mice were excluded from survival analysis because they did not show a lesion. An additional four mice were excluded from histology and behavioural analysis (three brains were damaged during the processing and one lesion size was an extreme outlier).

Mice in the two treatment groups received intraperitoneal injections of either vehicle control (saline) or 0.5 mg/kg fingolimod (obtained from Novartis Institutes for Biomedical Research, Basel). A researcher not associated with the study prepared the fingolimod solution in saline at a concentration of 0.06 mg/ml; the identity of the solution was hidden to the researcher carrying out the treatments. Male and female mice were randomly allocated to one of the two treatment groups using an online pseudo-random number generator (randomizer.org) a few days before surgery; the group allocation for each mouse was revealed at the time of first drug administration, i.e., 30 min after surgery (Figure 1). Subsequent dosing was administered 24 and 48 h later. Mouse husbandry, assessment of functional recovery and histological damage as well as data analysis were carried out in a blinded manner. Mice were kept for 14 days post-operatively. During that time they underwent daily monitoring, were weighed and allocated a neurological score as described (Bederson et al., 1986).

Due to the morbidity and mortality associated with this model, mice had to be added to the relevant groups in order to achieve a final n = 15 in each group; a total of 37 male and 54 female C57BL/6JOlaHsd mice (Envigo, United Kingdom) were used at 8–10 weeks of age. Three mice that showed no lesion were excluded from the analysis of histological/behavioural outcome and survival; one “outlier” mouse with extensive damage encompassing the whole hemisphere and three mice with incomplete datasets (missing behavioural assessment session or damaged tissue section) were excluded from the analysis of histology and behaviour (Figure 1C).

### Intracerebral Haemorrhage

We induced ICH by injecting collagenase VII-S (Sigma Aldrich, United Kingdom) into the striatum as previously described (Lu et al., 2014). Briefly, mice were anesthetized with 2% isoflurane in 100% O_2_ and placed in a stereotaxic frame (RWD Life Science). Temperature was maintained at 37°C during the procedure with a feedback thermoregulated surgical mat (NeosBiotec). A burr hole with 1 mm diameter was drilled 0.2 mm anterior and 2.0 mm lateral of bregma, on the right side of the skull. A blunt 26 G needle on a Hamilton syringe (2.5 ul, RN701) was inserted to a depth of 3.5 mm. Bacterial collagenase (0.075 U) in 0.5 μl of saline was injected, after which the needle was left in place for 10 min to prevent reflux up the needle track. The needle was slowly retracted, the burr hole covered with bone wax and the scalp sutured. Post-operative mice recovered for 30 min in a 32°C heated chamber.

### Behavioural Studies

We chose the cylinder, foot fault and the wire hanging tests to assess functional recovery. The first two tests evaluate the level of asymmetry associated with the unilateral damage to the striatum, while the wire hanging evaluates overall grip strength, balance, and endurance (Bermpohl et al., 2005; Balkaya et al., 2012).

The cylinder test was performed at baseline (before surgery), 6 and 13 days after collagenase injection (see Figure 1 for experimental protocol). Mice were placed in a glass cylinder and the vertical exploratory behaviour was recorded from above. The observer recorded for as long as it took for the mouse to complete 20 rears and wall touches. Recordings were analysed by an operator blinded to the treatment by counting the number of independent touches with ipsilateral (Right), contralateral (Left) or both (B) forepaws. Scores were calculated for each mouse as (R − L)/(R + L + B) as described (Balkaya et al., 2012).

Foot faults were assessed at baseline, and 7 and 14 days post-operatively. Mice were placed on a 25 × 35 cm wire grid with 1-cm^2^ openings and recorded while they walked from one end of the grid to the other. The first one hundred steps, and the number of times the ipsilateral or contralateral paw went through the grid missing the wire were counted. The score was calculated by the number of missed ipsilateral steps over contralateral ones (Balkaya et al., 2012).

Performance in the wire hanging test was assessed on the same days as the fault faults, after the respective foot fault testing session. Mice were placed on a wire midway between two poles, over a padded surface. Animals were scored based on their ability to hold on to the bar; a lowest score of 0 for failing to hold on to the wire up to a score of 5 for moving along the wire and going down the post (Bermpohl et al., 2005). The median score was calculated out of three attempts.

### Staining and Quantification of Lesion

Histological damage was assessed on brains collected 14 days post-ICH. Mice were euthanized by anaesthetic overdose and perfused transcardially with cold phosphate-buffered saline. The brains were frozen in 2-methylbutane at −40°C and covered in Shandon™ M-1 Embedding Matrix (Thermo Fisher). Coronal sections (20 µm thick) were taken at 500 µm intervals with a cryostat (Leica CM 1900 UV) at −20°C. Consecutive sections were stained with haematoxylin and eosin (H&E), and immunohistochemically with an antibody specific for nuclear neuronal protein (NeuN) (see below). The size of the lesions, hemispheric atrophy, and lateral ventricles were determined on both H&E and NeuN-stained sections scanned at 3,200 dpi with an Epson Perfection V550 scanner using ImageJ [v1.51f (Schneider et al., 2012)].

### Haematoxylin and Eosin

Slides with tissue sections were air dried overnight, fixed in 10% formalin for 5 min and then rehydrated in a series of graded alcohols, followed by 2 min in distilled water. The slides were then placed in Mayer's haematoxylin solution (Sigma) for 4 min, followed by rinsing in tap water until the water was clear and transferred in to 0.25% Eosin Y solution for 1 min. Lastly, slides were dehydrated through a series of alcohols, cleared in Histochoice (Sigma) and coverslipped with Permount mounting medium (Fisher Scientific).

### NeuN Immunohistochemistry

NeuN immunostaining was used because it has previously been shown to be a sensitive marker of neuronal loss from 1 day to 6 weeks after experimental stroke (Katchanov et al., 2003). Slides were air-dried, fixed for 20 min in methanol (Sigma) at −20°C. Peroxidase activity was blocked in a 10% H_2_O_2_ methanol solution for 10 min, followed by 1 h of blocking with 5% normal goat serum (S-1000, Vector Laboratories) in 0.3% Triton-X TBS at room temperature (RT). Tissue was incubated with anti-NeuN antibody (1:1,500, Abcam ab177487) for 1 h at RT, followed by 1 h incubation with goat anti-rabbit biotinylated secondary antibody (1:250, Abcam ab207995). Slides were washed 3 times with TBS, incubated in avidin–biotin horseradish peroxidase complex solution (ABC Kit pk-4000, Vector Laboratories) for 30 min and the reaction product visualized with diaminobenzidine (DAB Kit sk-4100, Vector Laboratories). Finally, the sections were lightly counterstained with eosin, dehydrated in alcohol, cleared with Histochoice, and the slides were cover-slipped with Permount mounting medium (Fisher Scientific).

### Lymphocyte Counts

The effect of fingolimod in the context of multiple sclerosis is thought to be due to a drastic reduction in the number of circulating lymphocytes (Brinkmann, 2009). In order to confirm that our fingolimod administration regimen was associated with a measurable effect in mice, we evaluated lymphocyte counts in a separate cohort of six male mice that were randomly treated with either saline or 0.5 mg/kg fingolimod in saline for 3 days. Blood was collected in EDTA tubes for FACS analysis. Red blood cells were lysed with 1X RBC lysis buffer (eBioscience). The samples were then incubated with the following fluorophore-conjugated antibodies at 4°C for 30 min: anti-mouse CD3ε PE-Cy-7 (Invitrogen, Clone 145-2C11), anti-mouse CD4 FITC (Invitrogen, Clone RM4-5) and anti-mouse CD8a PerCp-Cy5.5 (Invitrogen, Clone 53–6.7). All antibodies were used at a concentration estimated by titration experiments. Fixable Viability Dye eFluor 780 (eBioscience) was added as a live/dead cell stain and absolute cell counts were estimated using CountBright absolute counting beads (Invitrogen). Flow cytometric analysis was performed on a Becton Dickinson LSR II instrument, a minimum of 200,000 events were acquired for each sample. Data were analysed using FlowJo software (v8.6.3).

### Statistical Analysis

Mouse survival was visualized with Kaplan-Meier curves; the statistical significance of difference between groups was assessed using a Gehan-Breslow test. All data sets were evaluated with Shapiro-Wilk test to establish the data distribution and the appropriate statistical. Weights were analysed with a repeated measures mixed model. Histological measurements were analysed by a Mann Whitney test. Cylinder and foot fault test results were evaluated using repeated measures ANOVA. Wire hanging analysis was done with a Mann-Whitney test. Analysis was done with Prism 8 (GraphPad Software version 8.3.0). The significance level was set at *p* < 0.05.

## Results

### Fingolimod Improved Survival and Recovery in Female Mice

**FIGURE 2:**
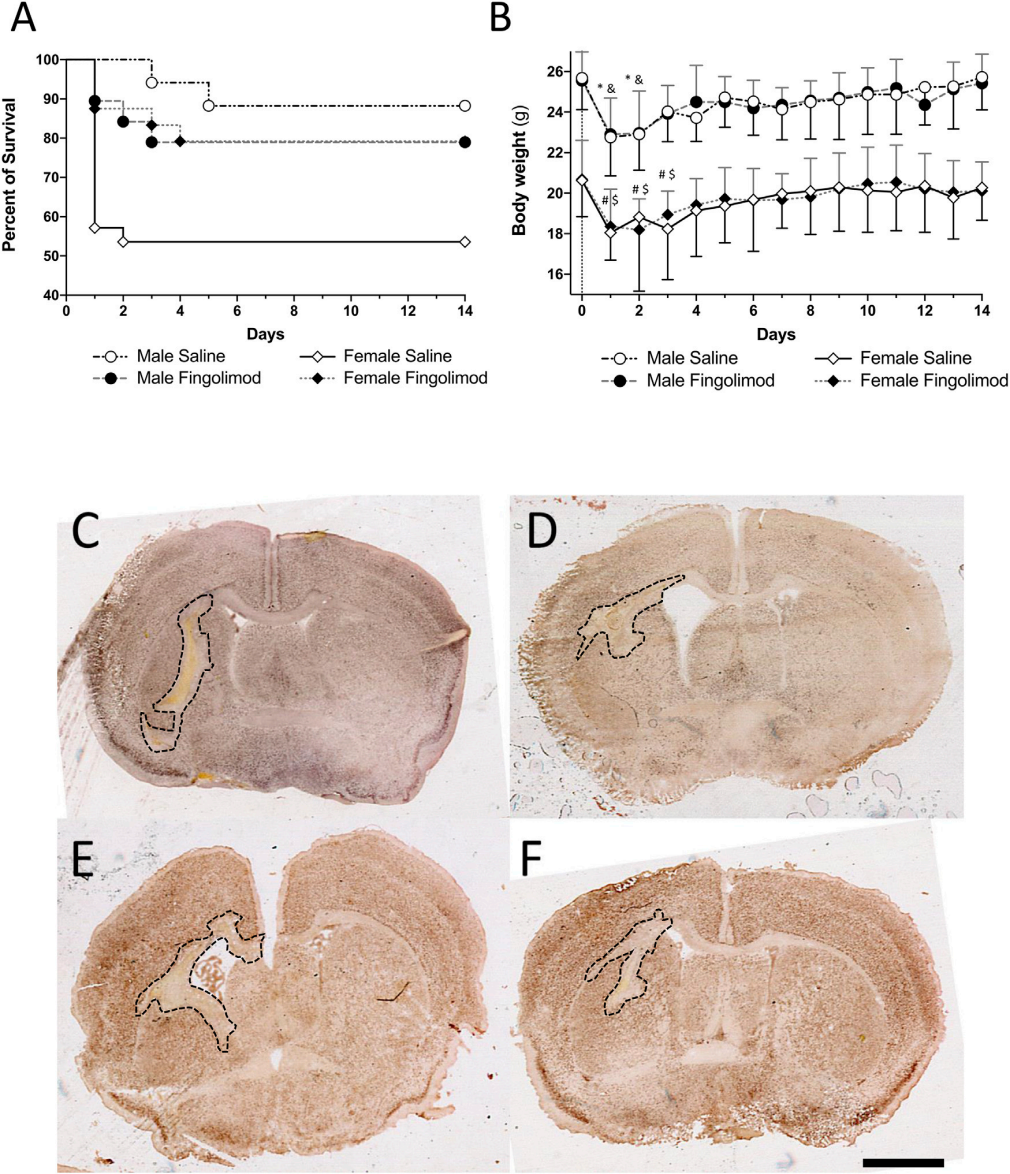
**(A)** Kaplan-Meir survival graph; female mice treated with fingolimod had a higher survival proportion than vehicle-treated mice and vehicle-treated male mice have a higher chance of survival than their female counterparts. **(B)** Weights over time shown as mean ± SD. Significant differences: * male saline compared to baseline; & male fingolimod compared to baseline; # female saline compared to baseline; $ female fingolimod compared to baseline. **(C–F)** Photomicrographs of NeuN stained section representative ICH-induced damage at 14 days in saline-**(A)** and fingolimod-treated male mice **(B)**, in saline-**(C)** and fingolimod-treated female mice **(D)**. All images were taken at the level of the anterior commissure. Lesions in all four tissue sections are outlined in black. Scale bar: 1 mm.

During the 14-day recovery period mice that had significant weight loss (>20%) and had a poor neuro-score were euthanised as it was unlikely that they would survive any further. The mice that were euthanised or were found dead during the observational period were recorded to assess the safety of fingolimod and the survival rate following the ICH procedure. The survival analysis (Figure 2A) showed a significant overall difference in survival between the groups (χ^2^ (3) = 10.88, *p* = 0.012). Male saline-treated (MS) mice had a significantly higher odds of survival than female saline-treated (FS) mice (χ^2^ (1) = 7.23, *p* = 0.007). However, there was no difference in survival between male fingolimod-treated (MF) and female fingolimod-treated (FF) mice (χ^2^ (1) = 0.001, *p* = 0.97). Female mice treated with fingolimod had a significantly higher odds of survival than saline treated female mice (χ^2^ (1) = 4.6, *p* = 0.032), but there was no survival difference between male mice treated with fingolimod or saline (χ^2^ (1) = 0.81, *p* = 0.37).

Male and female mice from both the treatment groups had a significant weight loss day one day after surgery when compared to baseline (MS: *p* = 0.0002; MF: *p* = 0.0003; FS: *p* < 0.0001; FF: *p* < 0.0001) (Figure 2B). After 3 days the weight of male mice was no longer significantly lower than baseline (MS: *p* > 0.99; MF: *p* = 0.36). It took one day longer for female mice to recover their baseline weight (at day 4, FS: *p* = 0.055; FF: *p* = 0.25). Treatment with fingolimod had no effect on weight loss or recovery.

### Fingolimod Does Not Decrease Lesion Volume and Tissue Loss

**FIGURE 3:**
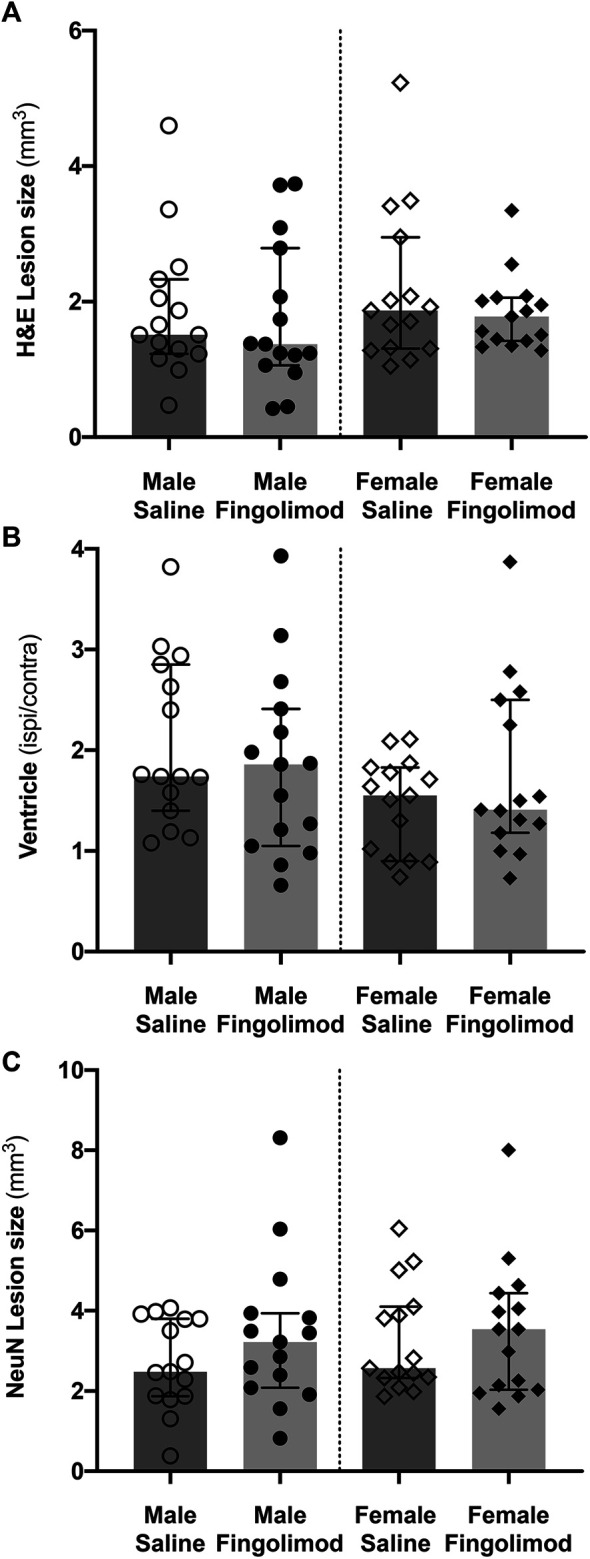
Quantification of histological damage. Lesion size was measured on haematoxylin/eosin-stained sections **(A)**, and NeuN-stained sections **(C)**. The ratio of ipsilateral to contralateral ventricle size was measured on haematoxylin/eosin-stained sections **(B)**. Values are shown as median ±95% confidence intervals. Fingolimod treatment had no significant effect on any of these measurements.

Fingolimod treatment showed no effect on lesion size in either male or female mice (Figures 2C–F). This was true whether the lesion size was measured on H&E-stained sections (Figure 3A) or on NeuN-stained sections (Figure 3C) (H&E, Male: *p* = 0.56, Female: *p* = 0.88; NeuN, Male: *p* = 0.41, Female: *p* > 0.99). Additionally, the ratio between the ipsilateral and contralateral ventricle sizes was measured as an estimate for the amount of tissue loss associated with ICH injury (Barratt et al., 2014). The ratio (measured on H&E stained sections) did not show any differences between the treatment groups (male: *p* = 0.51, female: *p* = 0.64) (Figure 3B). Sex as a variable did not have a significant effect on either lesion size or tissue loss, although saline-treated females showed a trend to a smaller ventricular enlargement, and hence lesser tissue loss, when compared to saline-treated males (*p* = 0.054).

### Fingolimod Does Not Improve Behavioral Outcome

**FIGURE 4:**
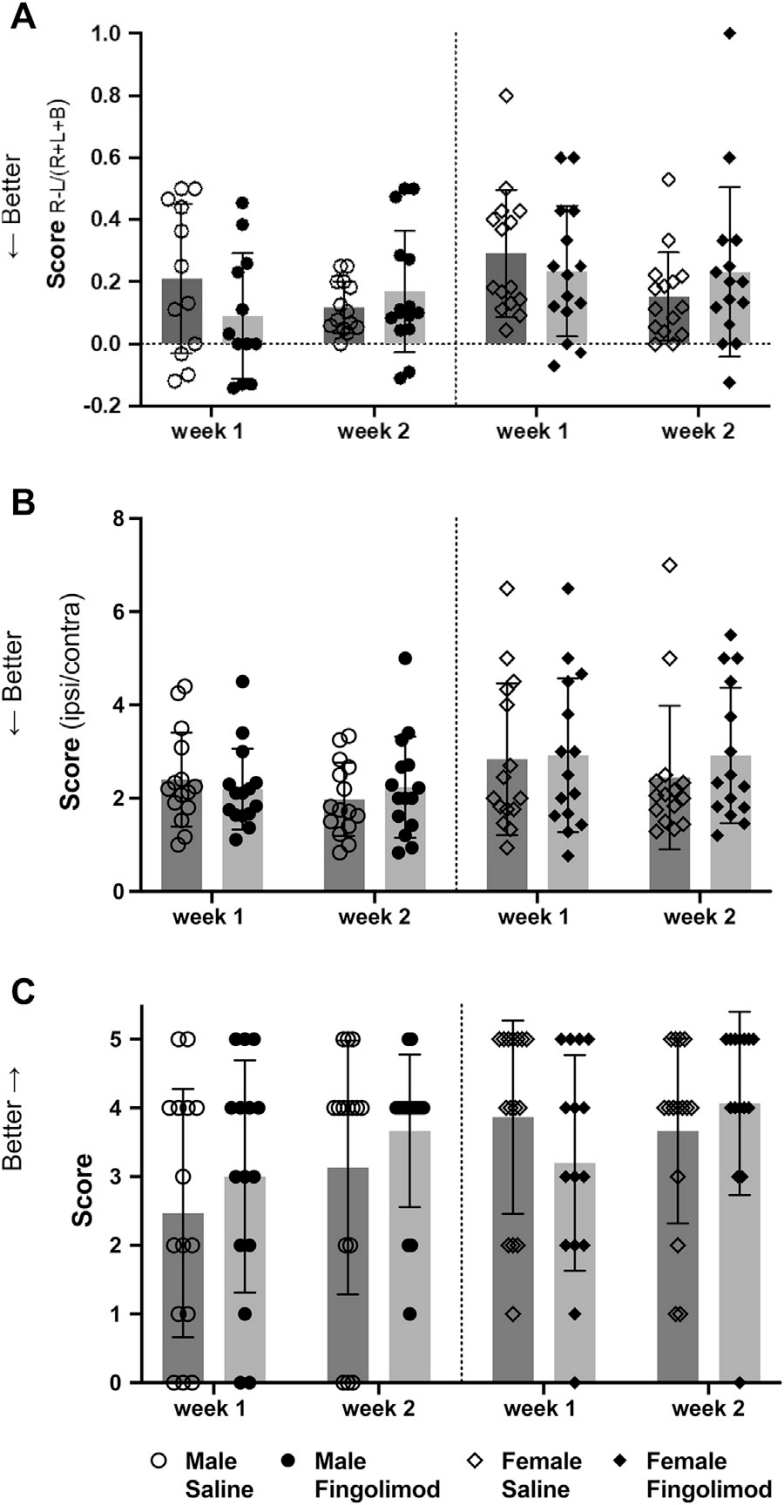
Behavioural test scores at two time points after intracerebral haemorrhage: (○) saline-treated males, (●) fingolimod-treated males, (◇) saline-treated females (◆) fingolimod-treated females). Mice were assessed using the cylinder test **(A)**: 6 and 13 days after collagenase injection. They were assessed using the foot fault test **(B)**: 7 and 14 days after collagenase injection. Finally, their performance in the wire hanging test **(C)**: was assessed on the same days as the foot fault test. Scores in **(A)** and **(B)** are shown as means ± SD, while wire hanging scores **(C)** are shown as medians ±95% confidence intervals. There was no difference between the scores of saline- and fingolimod-treated mice.

The cylinder test evaluated forelimb usage when exploring the sides of the cylinder. A score closer to 0 represents symmetrical limb usage, while a positive value represents a predominant usage of the unaffected ipsilateral forelimb (Figure 4A). A repeated measures ANOVA showed no effect of time (*p* = 0.92) or treatment (*p* = 0.51) on the recovery of male mice and no interaction between these variables (*p* = 0.08). Similarly, repeated measures ANOVA did not show significant difference in the recovery of the female mice (time: *p* = 0.062; treatment: *p* = 0.872; interaction: *p* = 0.073).

The foot fault test score evaluated the agility of the mice while crossing a grid. Lower ratios represent a better score (Figure 4B). A repeated measures ANOVA showed no effect of time (*p* = 0.31) or treatment (*p* = 0.93) on the recovery of male mice and no interaction between these variables (*p* = 0.23). Similarly, repeated measures ANOVA did not show significant difference in the recovery of the female mice (time: *p* = 0.57; treatment: *p* = 0.54; interaction: *p* = 0.58).

Lastly, the wire hanging test evaluated dexterity and the ability of the mice to use their forepaws to balance and walk off the wire (Figure 4C), with higher scores indicating better performance. There were no treatment-related differences in male mice after one (*p* = 0.43) or two weeks (*p* = 0.63), or in female mice at these time points (*p* = 0.22; *p* = 0.31).

### Fingolimod Decreases Circulating Lymphocyte Counts

**FIGURE 5:**
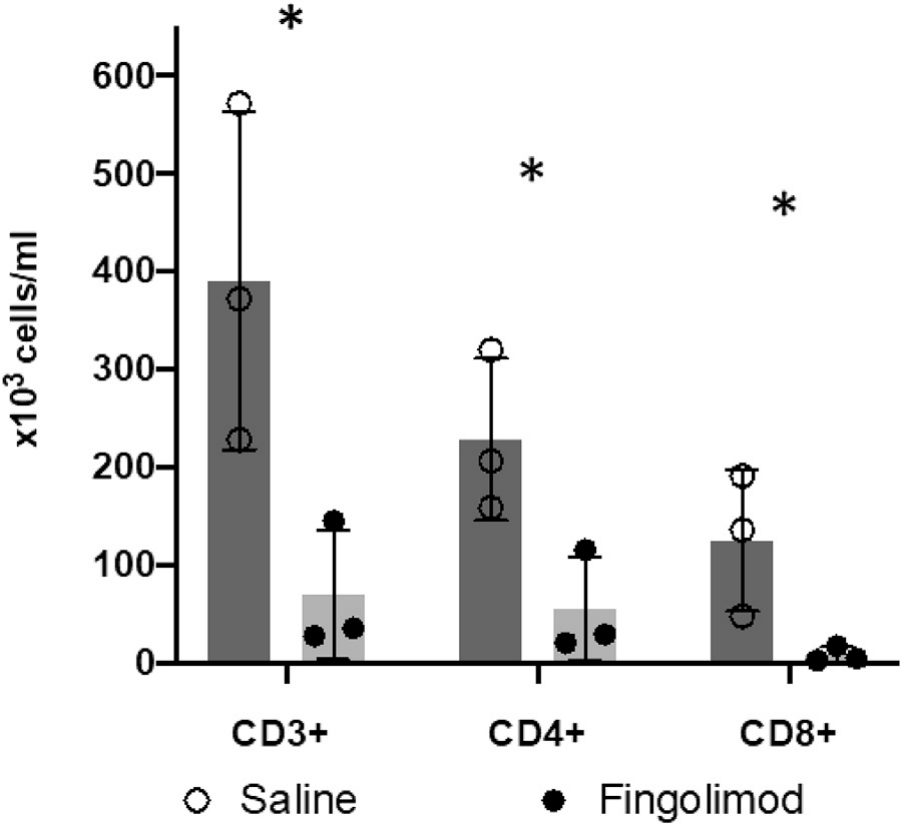
Fingolimod caused a significant drop in blood lymphocyte counts. (○) male saline-treated, (●) male fingolimod-treated mice (mean ± SD). Mice (n = 3 per group) treated for 3 days with 0.5 mg/kg of fingolimod had a significantly lower CD3-, CD4-and CD8-positive lymphocyte count than mice treated with saline; *p < 0.05.

To confirm drug delivery and the expected effect of fingolimod on circulating lymphocytes, we measured CD3-, CD4- and CD8-positive cells in blood samples (Figure 5). There was a significant difference in CD3^+^ cell counts between fingolimod and saline treated animals (82% reduction; *p* = 0.039). The CD4^+^ and CD8^+^ subpopulations were also significantly reduced by fingolimod, by 76% and 94% respectively (CD4^+^: *p* = 0.038; CD8^+^: *p* = 0.049).

## Discussion

The main finding of these studies is the lack of effect of fingolimod treatment on either histological or behavioural outcome measures after ICH. This contrasts with the significant effect of the same fingolimod administration regimen on circulating lymphocyte counts. To the best of our knowledge, three experimental ICH studies have reported a histological and/or functional improvement after treatment with fingolimod (Rolland et al., 2011; Rolland et al., 2013; Lu et al., 2014), with an additional study showing improvement following treatment with the more selective S1P receptor modulator siponimod (Bobinger et al., 2019) (Table 1). As mentioned in the introduction, there is much stronger evidence supporting a beneficial effect of fingolimod in experimental ischemic stroke, with 17 published articles (including 32 independent experiments) by a dozen different groups (Dang et al., 2020). It should however be noted that a small proportion of these published studies also failed to corroborate the effect of fingolimod in experimental ischemic stroke.

The current study was performed with an ICH model (collagenase injection) that was used in previous studies (Rolland et al., 2011; Rolland et al., 2013; Lu et al., 2014; Bobinger et al., 2019). The dose and administration regimen were similar to those previously found to be effective (Lu et al., 2014) although two studies used a higher dose of 1 mg/kg. It is unlikely that the use of a relatively lower dose accounted for the lack of effect in the present study, since this dose was sufficient to decrease the number of circulating T lymphocytes, but we cannot rule out that fingolimod exerts its effect via other mechanisms, unrelated to the adaptive immune system (Wang et al., 2020). It is important to mention that the outcome measures used to assess the effects of fingolimod in previous studies differed in part from the ones used here (Table 1) and that some of the behavioural measures often only showed a significant treatment effect on the first day after surgery. Previous studies with fingolimod used CD-1 mice. Since the immune response to brain injury varies between different rodent strains, it is possible that strain-related difference may account for the lack of effect of fingolimod in our study (Becker, 2016). Although siponimod was found effective in a collagenase injection model in C57BL/6 mice (Bobinger et al., 2019), while depleting circulating lymphocytes to the same extent, we cannot rule out the effect of fingolimod is more dependent on mouse strain than the effect of siponimod. Furthermore, since C57BL/6 mouse substrains show different vulnerability to ischemic stroke (Zhao et al., 2019), it remains possible that they also show different sensitivity to the effect of S1P receptor modulators in ICH.

One of the drawbacks of the collagenase model of ICH is due to the ability of this enzyme to induce a significant inflammatory reaction (Manaenko et al., 2011; Barratt et al., 2014), which might be exacerbated by the possible presence of endotoxin or other contaminants in the collagenase preparation (Jahr et al., 1999; Nomura et al., 2017). It is possible that inhibition of endotoxin-mediated activation of microglial cells may have accounted for some of the effects of fingolimod in collagenase-based ICH models (Wei et al., 2011; Lu et al., 2014), but it is worth pointing out that fingolimod was also found to be effective in a mouse ICH model based on autologous blood injection (Rolland et al., 2013). Another drawback of this study and of previous studies of S1P receptor agonists in ICH is that fingolimod or siponimod were administered for at most 3 days after surgery. It is therefore impossible to rule out the possibility that treating animals for the whole duration of the study (i.e., 14 days or more) would have shown a treatment effect.

It is possible that we failed to detect a true effect of fingolimod (Type II error), or that the conclusions drawn from previous studies were due to Type I errors. As mentioned, our *a priori* power calculation for this ICH study was based on the effect sizes and variability seen in the ischemic stroke literature on the effects of fingolimod (Liu et al., 2012). In retrospect, we may have underestimated the variability of the outcome measures assessed in this study, and hence the group sizes required to detect an improved outcome after fingolimod treatment. However, considering the lack of even a trend toward improvement in our data, we considered that it would have unethical to adjust group sizes and add more mice to our study. Published studies have in common relatively smaller group sizes and/or the lack of proper power calculation (Table 1). This observation, taken together with the notion that low statistical power is more likely to be associated with false positives, would be compatible with the possibility that previous findings of a beneficial effect of fingolimod in experimental ICH may not have reflected a true effect (Ioannidis, 2005; Button et al., 2013).

Two of the previous studies of fingolimod in experimental ICH did not report mortality (Rolland et al., 2011; Rolland et al., 2013), while only one mouse died, in the vehicle-treated group, in the third study (Lu et al., 2014). Neither these studies nor a more recent one using siponimod (Bobinger et al., 2019) included female mice. Although we did not set out to study possible sex-related differences in the response to fingolimod, and therefore did not power our study to detect an effect of sex on outcome measures, it is difficult to ignore the observation that female mice, studied concurrently with male mice, had a significantly higher mortality than male mice, and regained weight more slowly than male mice. The vast majority of experimental ICH studies are performed in male mice (Kirkman et al., 2011; Liddle et al., 2020) and we are not aware of previous studies showing a poorer outcome in female rodents. In fact, in an autologous blood injection model, female mice showed significantly less oedema and their behavioural deficits recovered faster compared to male mice (Nakamura et al., 2004). Similar results were reported using the collagenase injection model (Lei et al., 2012; Xie et al., 2019). Another report showed similar brain injury in male and female mice following autologous blood injection, although only a small number of female mice were studied, and no direct comparison was performed (Jing et al., 2019). Clinically, most epidemiological studies either show no sex difference in ICH incidence, or higher incidence in men (Gokhale et al., 2015). Similarly, no significant sex differences in ICH mortality have been observed in most studies, while some studies show a higher age-adjusted mortality in men (Gokhale et al., 2015). There is however evidence that female sex may be associated with poorer neurological outcome either early following ICH or later on (Ganti et al., 2013; Umeano et al., 2013). Interestingly, our study shows a significantly improved survival in female mice treated with fingolimod. Despite our failure to observe a drug effect on histological and behavioural outcomes, this may suggest that fingolimod may have a beneficial effect after ICH, specifically when it is associated with a worse outcome.

In conclusion, these studies reinforce the need to perform rigorous and well powered experimental ICH studies, following the STAIR and RIGOR guidelines (Liddle et al., 2020), and to report them according to the ARRIVE guidelines. Although the absence of effect of fingolimod in the present study seems to contradict the results of previous preclinical studies, it does not invalidate the hypothesis that S1P receptor modulators may be effective in ischemic and haemorrhagic stroke. In fact, a small proof of concept clinical trial points to an efficacy of these agents for both conditions (Fu et al., 2014a; Fu et al., 2014b; Zhu et al., 2015; Tian et al., 2018). Further animal studies are therefore required to determine whether fingolimod and/or siponimod are effective in specific populations (males vs females, aged animals or animals with typical stroke comorbidities such as hypertension or hyperlipidaemia) and using different treatment regimens (longer durations and/or higher dose). Similarly, at the clinical levels, larger trials will be needed to confirm the effects seen in ICH patients.

## Data Availability Statement

The original contributions presented in the study are included in the article, further inquiries can be directed to the corresponding author.

## Ethics Statement

The study was performed following approval by the Animal Experimentation Ethics Committee (AEEC) at University College Cork, under a project authorization by the Irish Health Products Regulatory Authority (HPRA; AE19130/P042).

## Author Contributions

All authors contributed to the drafting of the manuscript. In addition, ADD performed the rodent surgery, behavioural and statistical analysis, JS performed the histological staining and lesion size measurements, KM performed the flow cytometry studies, CW conceived and supervised the studies and finalized the manuscript.

## Funding

These studies were funded by a Health Research Award (HRA-POR-2015-1236) from the Health Research Board (HRB) of Ireland.

## Conflict of Interest

The authors declare that the research was conducted in the absence of any commercial or financial relationships that could be construed as a potential conflict of interest.
